# Introduction to Special Issue: Plant Microbiome Augmentation and Stimulation—New Strategies to Grow Crops with Reduced Agrochemicals

**DOI:** 10.3390/microorganisms9091887

**Published:** 2021-09-06

**Authors:** Miguel J. Beltran-Garcia, James F. White

**Affiliations:** 1 Lab 309-E Building, Chemistry Department, Universidad Autonoma de Guadalajara, Zapopan 45129, Jalisco, Mexico; 2 Departamento de Biotecnologicas y Ambientales, Universidad Autonoma de Guadalajara, Zapopan 45129, Jalisco, Mexico; 3 Department of Plant Biology, Rutgers University, New Brunswick, NJ 08901, USA

Since the early work of Justus von Liebig on nutrient absorption in plants in the 1800s [1], industrial agriculture has been trapped in a ‘chemical paradigm’ that holds that agrochemicals (inorganic fertilizers, pesticides, fungicides, and herbicides) are essential to producing healthy crops and increase crop yields. The ways in which we have treated soils and seeds with disregard for microbes have decimated native microbial communities in plants and soils and inhibited the function of the microbe-based systems plants use to obtain nutrients, adapt to stress, and control pathogens and other pests. Consequently, our crops have become increasingly dependent on agrochemicals for cultivation and control of diseases and pests. Much of the agricultural research conducted over the years has only served to reinforce the widespread belief of the essentialness of agrochemistry in our decimated plant cultivation systems. The use of microbes by non-cultivated plants in natural environments has been largely overlooked by scientists and agriculturists alike due to our agrochemical myopia. However, it is evident that non-cultivated plants do not require agrochemicals at all; rather, plants in natural settings use microbes in their tissues and in soils to acquire nutrients, maintain health, and control diseases and pests. A picture is now emerging that suggests that it may be possible to cultivate crops utilizing largely plant and soil microbes rather than agrochemicals [2,3]. In order to grow crops, plant microbiomes may be augmented with microbes that function in plants—perhaps acquired from wild relatives of crop plants [2]. Another strategy is to use substances (e.g., humic substances, algal extracts, ferments, etc.) that stimulate or activate the microbiome-associated systems (e.g., rhizophagy cycle) in plants to function optimally [3,4]. In this Special Issue we have assembled a selection of papers that highlight how the plant and soil microbiomes may be used to cultivate crop plants. 

In this Special Issue, two articles provide data and discuss the roles of microbes in attaining sustainability in agricultural production. Among these articles, Beltran-Garcia et al. [5] discuss use of probiotic microbes to attain sustainability in banana production. Kustatscher et al. [6] show how microbiome augmentation may be used in combination with fungicides to enhance plant health. Two articles deal with the roles of microbes in delivering nutrients to plants. Among these, Chang et al. [7] show how intracellular endophytes in root hairs interact chemically with the root cell to cause root hair elongation, deliver nitrogen to the plant, and increase plant hardiness. Vaitiekūnaitė et al. [8] discuss and evaluate the use of microbes as biofertilizers in *Populus* spp. Two articles address the use of microbes to enhance abiotic stress tolerance in plants. Among these, Molina et al. [9] describe an in vitro gnotobiotic model to evaluate contributions of endophytic microbes in the enhancement of plant survival under arsenic stress. Mahdi et al. [10] show how microbes may be used to increase salt stress tolerance in plants. Biological control based on beneficial bacteria and fungi substantially reduce pathogen susceptibility and plant disease incidence. However, there are still limitations that affect crop protection. In this Special Issue, several articles deal with the use of microbes to control plant disease agents or pests. These papers include ones by Toral et al. [11] and Castro et al. [12] that show how *Bacillus velezensis* strains may be used to control fungal disease incited by *Botrytis cinerea* in tomato and *Verticillium* wilt in olives. Marsico et al. [13] describe the use of yeasts to control postharvest *Botrytis* disease in grapes. Finally, Leoni et al. [14] evaluate the use of nematocidal fungus in tomato plants to control root-knot nematodes.

This collection of articles provides a sampling of work that suggests that we may develop a more balanced ‘biological future’ for industrial plant agriculture.
